# Prognostic role of programmed-death ligand 1 (PD-L1) expressing tumor infiltrating lymphocytes in testicular germ cell tumors

**DOI:** 10.18632/oncotarget.15585

**Published:** 2017-02-21

**Authors:** Michal Chovanec, Zuzana Cierna, Viera Miskovska, Katarina Machalekova, Daniela Svetlovska, Katarina Kalavska, Katarina Rejlekova, Stanislav Spanik, Karol Kajo, Pavel Babal, Jozef Mardiak, Michal Mego

**Affiliations:** ^1^ 2nd Department of Oncology, Comenius University, Faculty of Medicine & National Cancer Institute, Bratislava, Slovak Republic; ^2^ Translational Research Unit, 2nd Department of Oncology, Comenius University, Faculty of Medicine & National Cancer Institute, Bratislava, Slovak Republic; ^3^ Department of Pathology, Comenius University, Faculty of Medicine, Bratislava, Slovak Republic; ^4^ 1st Department of Oncology, Comenius University, Faculty of Medicine & St. Elisabeth Cancer Institute, Bratislava, Slovak Republic; ^5^ Department of Pathology, Slovak Medical University St. Elisabeth Cancer Institute, Bratislava, Slovak Republic; ^6^ Department of Clinical Trials, National Cancer Institute, Bratislava, Slovak Republic; ^7^ Department of Medical Oncology, National Cancer Institute, Bratislava, Slovak Republic; ^8^ Cancer Research Institute, Slovak Academy of Sciences, Bratislava, Slovak Republic; ^9^ Faculty Hospital with Policlinics Skalica, a.s., Skalica, Slovak Republic

**Keywords:** programmed death-ligand 1, programmed cell death protein 1, tumor infiltrating lymphocytes, prognostic factor, testicular germ cell tumors

## Abstract

**Purpose:**

Testicular germ cell tumors (TGCTs) are nearly universally curable malignancies. Nevertheless, standard cisplatin-based chemotherapy is not curative in a small subgroup of patients. Previously, we showed that PD-L1 overexpression is associated with worse prognosis in TGCTs, while tumor infiltrating lymphocytes (TILs) are prognostic in different types of cancer. This study aimed to evaluate the prognostic value of PD-1 and PD-L1 expressing TILs in TGCTs.

**Results:**

PD-L1 positive TILs were found significantly more often in seminomas (95.9% of patients) and embryonal carcinomas (91.0%) compared to yolk sac tumors (60.0%), choriocarcinomas (54.5%) or teratomas (35.7%) (All p < 0.05). TGCTs patients with high infiltration of PD-L1 positive TILs (HS ≥ 160) had significantly better progression-free survival (HR = 0.17, 95% CI 0.09 – 0.31, p = 0.0006) and overall survival (HR = 0.08, 95% CI 0.04 – 0.16, p = 0.001) opposite to patients with lower expression of PD-L1 (HS < 150). PD-1 expressing TILs were not prognostic in TGCTs.

**Materials and Methods:**

Surgical specimens from 240 patients with primary TGCTs were included into this translational study. The PD-1 and PD-L1 expression on tumor and TILs were detected by immunohistochemistry using anti-PD-1 and anti-PD-L1 monoclonal antibody. Scoring was performed semiquantitatively by weighted histoscore (HS) method.

**Conclusions:**

The prognostic value of PD-L1 expressing TILs in TGCTs was demonstrated for the first time.

## INTRODUCTION

Testicular germ cell tumors (TGCTs) are nearly universally curable malignancies, most commonly found in young men [1]. Cisplatin based chemotherapy is curative for 70-80% of patients with metastatic disease, although 20-30% of them may suffer from relapse [2, 3]. Subsequent salvage chemotherapy may cure only 20-25% of patients with relapsed TGCTs [3–5]. The prognosis of patients who failed to be cured with conventional treatment is dismal. Therefore our interest needs to be directed towards identification of predictive biomarkers and novel treatment strategies [6].

Malignant tumor biology and its’ associations with immune mechanisms have been recently a subject of intensified research. It occurs that immune check-point inhibition including PD-1, PD-L1 and/or CTLA4 is a highly effective modality in several types of malignancies [7–10]. Tumor infiltrating lymphocytes (TILs) are likewise proven to be an important biomarker with strong prognostic and predictive features in different types of tumors [11–13].

Programmed-death-1 receptor (PD-1; CD279) and its ligand (PD-L1; B7-H1; CD274) are promising biomarkers and treatment targets in various types of tumors. PD-1 and PD-L1 deliver inhibitory signals that regulate balance between T-cell activation, tolerance and immune-mediated tissue damage [14]. PD-L1 and PD-L2 are ligands primarily involved in modulating T cell activity in peripheral tissues through interaction with PD-1 receptor [15]. PD-1 is a member of the immunoglobulin superfamily and is expressed on double negative T cells in thymus and on activated CD4^+^ T cells, CD8^+^ T cells, natural killer cells, B cells and monocytes [16]. PD-L1 is expressed in different organs, including placenta, heart, lung and liver as well as on activated T cells, B cells, dendritic cells, macrophages, mesenchymal stem cells and subsets of thymocytes. PD-L2 expression was not observed in the lymphohematopoietic cells at all [16–18]. Expression of PD-L1 was also described in various tumor cells [16]. Tumor cells are able to suppress antitumor immunity through PD-L1 signaling in the tumor microenvironment [19]. Prognostic significance of PD-L1 expression in tumor was described in various malignancies, including non-small cell lung cancer [8, 9], malignant melanoma [8, 10], renal cell carcinoma [8, 20], colorectal [21] breast cancer [22] and testicular cancer [23].

The presence of TILs have been proven to have clear correlations with patients’ clinical outcome in several types of tumors, such as metastatic melanoma [24], ovarian [25, 26], colorectal [13] and breast cancer [11, 27]. TILs in breast cancer is a robust predictive and prognostic marker [28] and their existence in breast cancer tissue before chemotherapy seems to be a good prognostic feature promoting a therapeutic effect of neoadjuvant treatment [29, 30].

Previously we have shown that PD-L1 expression in cancer cells have a prognostic role in TGCTs [23]. However, expression of PD-L1 on TILs in TGCTs was not reported to this date. The aim of this translational study was to evaluate the expression of PD-1 and PD-L1 in TILs in TGCTs and to investigate their possible prognostic value.

## RESULTS

### Patients’ characteristics

Patients’ characteristics are summarized in Table 1. Majority of patients had non-seminomatous primary TGCT, and a good prognosis according to IGCCCG. Patients were managed with chemotherapy, radiotherapy or surveillance. Tumor specimens from 240 patients before administration of systemic therapy included 57 pure seminomas and 183 non-seminomas/mixed GCTs. Non-seminomas and mixed GCTs comprised of 140 embryonal carcinomas, 24 seminomas, 26 yolk sac tumors, 13 choriocarcinomas and 36 teratomas (Table 2). 9 cases of seminomas were clinically considered as non-seminomas based on positivity of alpha-fetoprotein.

**Table 1:** Patients’ characteristics (n = 240)

	N = 240	%
**Age (years)**		
Median (range)	30,6 (16-67)	
**Histology**		
Pure seminoma	57	23.8
Non-seminoma or mixed TGCTs	183	76.3
**Primary tumor**		
Gonadal	240	100.0
**IGCCCG risk group**		
Good risk	184	76.7
Intermediate risk	27	11.2
Poor risk	29	12.1
**Sites of metastases**		
Retroperitoneum	167	69.6
Mediastinum	27	11.3
Lungs	54	22.5
Liver	14	5.8
Other	35	14.6
Non-pulmonary visceral metastases	17	7.1
**No. of metastatic sites**		
0	65	27.1
1	104	43.3
2	33	13.8
> 3	38	15.8
**Mean (range) of pretreatment markers**		
AFP mIU/ml	998 (0-60570)	
β-HCG IU/ml	10633 (0-423338)	
LDH (mkat/l)	12 (1.97-89)	

**Table 2:** Distribution of histological subtypes among TGCT patients (n=240)

Histological subtype					Number of patients
	SEM				57
EC					94
				TER	12
		YST			21
			ChC		4
EC	SEM		ChC		1
	SEM			TER	2
EC	SEM			TER	2
	SEM	YST			2
EC	SEM				17
EC		YST			3
EC				TER	15
EC			ChC	TER	4
EC			ChC		4
		YST	ChC	TER	1
		YST		TER	1

Abbreviations: EC, embryonal carcinoma; SEM, seminoma; YST, yolk sac tumor; ChC, choriocarcinoma; TER, teratoma

### Distribution of PD-L1 and PD-1 positive TILs among histological subtypes

TILs were not found in specimens from 25 of 240 patients (10.4%) in this cohort (Supplementary Figure 1). TILs from 14 of 240 patients (5.8%) did not express PD-1 nor PD-L1. PD-L1 positive TILs were found significantly more often in seminomas (95.9% of all 225 patients with TILs) and embryonal carcinomas (91.0%) compared to yolk sac tumors (60.0%), choriocarcinomas (54.5%), teratomas (35.7%) or germ cell neoplasia *in situ* (GCNIS) (80.6%) (All p < 0.05). Similarly, the highest percentage of PD-L1 expressing TILs was found in seminoma (61.0% of all TILs), followed by embryonal carcinoma (42.4%), yolk sac tumor (37.9%), teratoma (24.2%) and choriocarcinoma (21.4%) while 36.5% of lymphocytes found between tubules of GCNIS expressed PD-L1.

The expression of PD-L1 on TILs differed significantly across distinct histological subtypes. Seminoma had significantly higher PD-L1 expression on TILs compared to embryonal carcinoma (p = 0.002), choriocarcinoma (p = 0.0001), yolk sac tumor (p = 0.0001) and teratoma (p < 0.0001) (Table 3). When we dichotomized PD-L1 expression on TILs, 37.0% of seminomas, 19.3 % of embryonal carcinomas and 19.2 % of yolk sac tumors had high PD-L1 expression (HS ≥ 160), while high PD-L1 expression on TILs was uncommon in teratomas (2.8%) and completely missing in choriocarcinomas (Table 3).

**Table 3:** PD-L1 expression on TILs in different histologic subtypes of primary testicular germ cell tumors (n = 225)*

Histologic subtype	N	PD-L1 expression on TILs								
		Mean Score	SEM	Median	p-value ^a^	Low ^b^		High ^c^		p-value ^a^
						N	%	N	%	
Seminoma	81	136.80	11.71	100	NA	51	63.0	30	37.0	NA
GCNIS	75	63.68	11.23	30	<0.001	64	86.5	11	14.9	0.002
Embryonal carcinoma	140	89.00	8.34	60	0.002	113	80.7	27	19.3	0.006
Yolc sac tumor	26	56.37	21.06	2.5	<0.001	21	80.8	5	19.2	0.015
Choriocarcinoma	13	20.77	28.43	0	<0.001	13	100.0	0	00.0	0.008
Teratoma	36	10.42	15.85	0	<0.001	35	97.2	1	02.8	<0.001

* 225 of 240 patients had TILs in tumor specimen

Abbreviations: HS - multiplicative quickscore; SEM – standard error of the mean; NA – not applicable

a compared to seminoma

b HS 0 – 150

c HS 160 – 300

PD-1 expressing TILs were found in 50 of 57 patients (87.7%) with pure seminoma or seminomatous component in TGCTs. Six of 50 patients (12%) with seminoma had high PD-1 expression on TILs in contrast to 44 patients (88%) with low expression. PD-1 expressing TILs were found in 60 of 140 patients (42.9%) with embryonal carcinoma component, only 1 patient of 60 (1.7%) had high PD-1 expression. PD-1 positive TILs were present in 14 of 36 patients (38.8%) with teratoma and 7 of 26 patients (26.9%) with yolk sac tumor, all with low expression. None of choriocarcinoma patients had PD-1 expression on TILs.

### Association between PD-L1 positive TILs and patients/tumor characteristics

Table 4 shows exploratory analysis of associations between patients/tumor characteristics and PD-L1 expressing TILs in TGCTs. Pure seminomas had significantly higher expression of PD-L1 on TILs compared to non-seminomas and mixed TGCTs (p = 0.0009). Lower expression of PD-L1 on TILs was associated with poor prognosis according to IGCCCG (p < 0.0001). Significantly lower expression of PD-L1 on TILs was seen in patients with more than 3 metastatic sites, mediastinal lymphadenopathy and non-pulmonary visceral metastases (NPVM). S3 stage was associated with lower expression of PD-L1 on TILs as well.

**Table 4:** Patient's characteristics according to PD-L1 expressing TILs in TGCTs (n = 240)

Variable	N	PD-L1 expression								
		Mean score	SEM	Median	p-value	Low ^a^		High ^b^		p-value
						N	%	N	%	
**All patients**	240	99.40	6.73	60	NA	179	74.6	61	25.4	NA
**Histology**										
Pure seminoma	48	143.54	14.73	140	0.0009	30	62.5	18	37.5	0.040
Non-seminoma or mixed TGCTs	192	88.36	7.36	45		149	77.6	43	22.4	
**IGCCCG risk group**										
Good and intermediate risk	211	108.93	6.96	100	<0.0001	151	71.6	60	28.4	0.002
Poor risk	29	30.03	18.79	0		28	96.6	1	3.4	
**Number of metastatic sites**										
0 to 2	203	103.37	7.30	60	0.04	150	73.9	53	26.1	0.680
≥ 3	37	77.59	17.10	10		29	78.4	8	21.6	
**Retroperitoneal LN metastases**										
Absent	73	89.11	12.19	50	0.36	55	75.3	18	24.7	1.000
Present	167	103.90	8.06	60		124	74.3	43	25.7	
**Mediastinal LN metastases**										
Absent	213	104.32	7.09	90	0.01	157	73.7	56	26.3	0.490
Present	27	60.59	19.92	5		22	81.5	5	18.5	
**Lung metastases**										
Absent	186	101.91	7.65	60	0.54	137	73.7	49	26.3	0.600
Present	54	90.76	14.19	40		42	77.8	12	22.2	
**Non-pulmonary visceral metastases**										
Absent	223	102.72	6.95	60	0.03	164	73.5	59	26.5	0.250
Present	17	55.88	25.15	5		15	88.2	2	11.8	
**S – stage**										
0-2	220	105.95	6.89	90	0.0004	160	72.7	60	27.3	0.030
3	20	27.30	22.83	0		19	95.0	1	5.0	

Abbreviations: LN – lymph nodes, SEM – standard error of the mean, NA – not applicable, ^a^ HS 0 – 150 ^b^ HS 160 – 300

### Association between PD-L1 positive TILs and patient outcome

The median follow-up was 78.9 months (0.4-293.7 months) for all patients and 120.7 months (9.5-293.7 months) for patients still alive. During the follow-up period, 50 (20.8%) patients experienced disease progression and 35 (14.6%) patients died. The estimated 5-year PFS and OS for all patients was 80.8% (95% CI 75.8% to 85.9%) and 86.8% (95% CI 82.4% to 91.1%), respectively.

**Figure 1:**
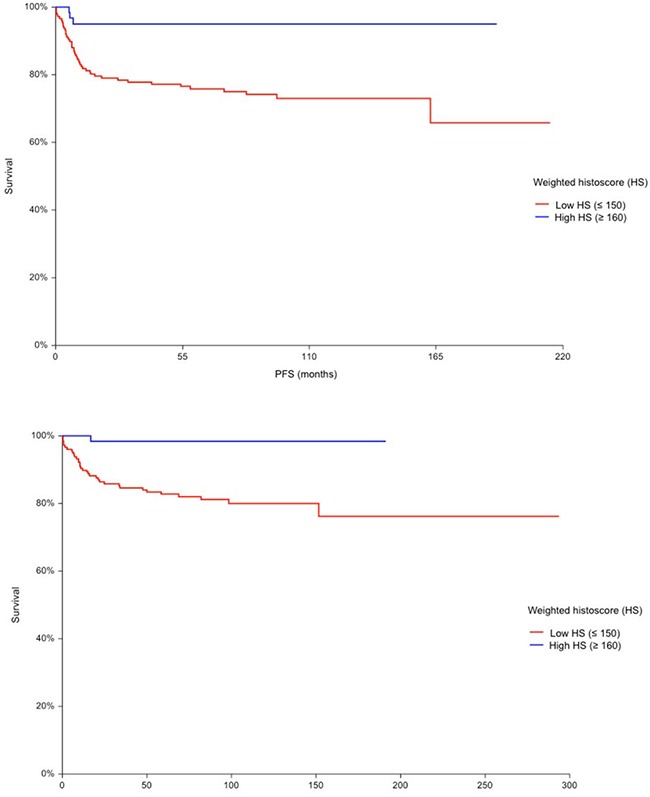
**A.** Kaplan-Meier estimates of probabilities of progression-free survival according to PD-L1 expressing tumor infiltrating lymphocytes (TILs); in patients with primary TGCTs (n = 240), Hazard ratio = 0.17, 95% CI 0.09 – 0.31, p = 0.0006, Low HS - low PD-L1 expressing TILs; High HS- high PD-L1 expressing TILs. **B.** Kaplan-Meier estimates of probabilities of overall survival according to PD-L1 expressing tumor infiltrating lymphocytes (TILs); in patients with primary TGCTs (n = 240), Hazard ratio = 0,08, 95% CI (0.04 – 0.16), p = 0.001, Low HS - low PD-L1 expressing TILs; High HS- high PD-L1 expressing TILs.

TGCTs with high expression of PD-L1 on TILs (HS ≥ 160) had significantly better PFS (HR = 0.17 95% CI 0.09 – 0.31, p = 0.0006) (Figure 1A) and OS (HR = 0.08, 95% CI 0.04 – 0.16, p = 0.001) (Figure 1B) opposite to patients with low expression of PD-L1 (HS ≤ 150) on TILs. PD-L1 expression on TILs was associated with PFS and OS independently of IGCCCG risk group in multivariable analysis (Table 5). Results of multivariable analysis were confirmed when we used IGCCCG in three categories and expression of PD-L1 as a continuous variable (data no shown). PD-1 expressing TILs were not found to be prognostic in TGCTs.

**Table 5:** Multivariable analysis

Variable	PFS		OS	
	HR (95% C.I.)	*P* – value	HR (95% C.I.)	*P* – value
**PD-L1 expressing TILs in primary tumor** High vs. Low	0.2224(0.0683 - 0.7248)	0.0126	0.1186(0.0160 - 0.8809)	0.0372
**IGCCCG risk group** Poor vs. Good/intermediate risk group	4.8008(2.6363 - 8.7423)	<0.0001	6.4267(3.2747 - 12.6124)	<0.0001

**Figure 2:**
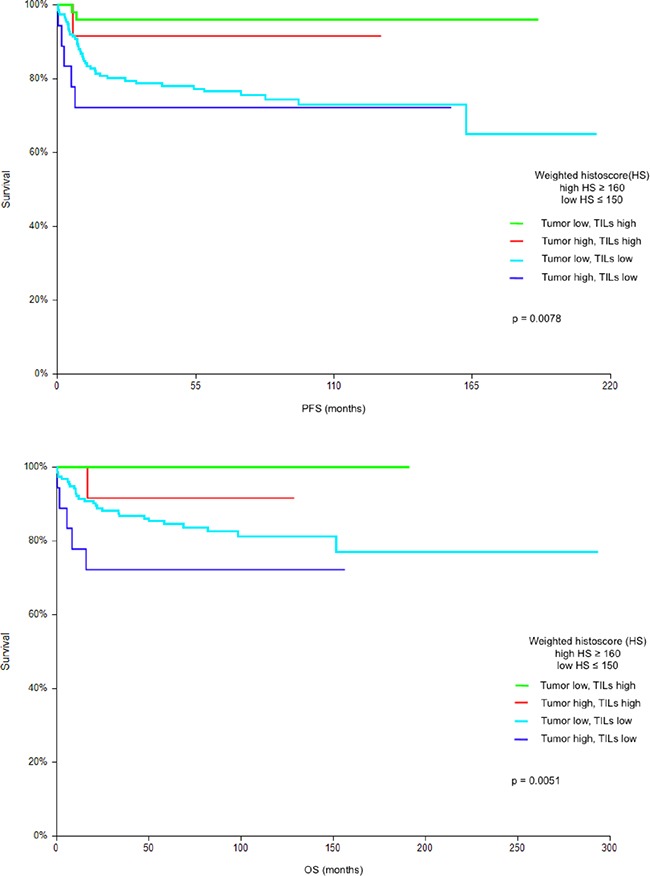
**A.** Kaplan-Meier estimates of probabilities of progression-free survival according to PD-L1 expression on tumor cells and tumor infiltrating lymphocytes (TILs); in patients with primary TGCTs (n = 240), Low HS - low PD-L1 expression; High HS- high PD-L1 expression. A 5-year progression-free survival in groups of patients according to PD-L1 expression in tumor and TILs were as follows: low PD-L1 in tumor and high PD-L1 on TILs: 95.9%; high PD-L1 in tumor and high PD-L1 on TILs: 91.7%; low PD-L1 in tumor and low PD-L1 on TILs: 76.5 %; high PD-L1 in tumor and low PD-L1 on TILs: 72.2%. **B.** Kaplan-Meier estimates of probabilities of overall survival according to PD-L1 expression on tumor cells and tumor infiltrating lymphocytes (TILs); in patients with primary TGCTs (n = 240), Low HS - low PD-L1 expression; High HS- high PD-L1 expression. A 5-year overall survival in groups of patients according to PD-L1 expression in tumor and TILs were as follows: low PD-L1 in tumor and high PD-L1 on TILs: 100%; high PD-L1 in tumor and high PD-L1 on TILs: 91.7%; low PD-L1 in tumor and low PD-L1 on TILs: 84.5 %; high PD-L1 in tumor and low PD-L1 on TILs :72.2%.

In subsequent analysis we assessed combined prognostic value of PD-L1expression on tumor cells and TILs. The analysis revealed four distinct groups of patients with significant differences in PFS and OS. Patients with low expression of PD-L1 in tumor and high expression of PD-L1 on TILs had the best prognosis with 5-year PFS and OS 95.9% and 100%, respectively, while patients with high expression of PD-L1 in tumor and low expression of PD-L1 on TILs had the worst prognosis with 5-year PFS and OS 72.2% and 72.2%, respectively (HR = 0.13, p = 0.0078 for PFS and HR < 0.01, p = 0.0051 for OS). (Figure 2).

## DISCUSSION

This translational study have shown significant differences in expression of PD-L1 on TILs in distinct histological subtypes of TGCTs. The prognostic value of PD-L1 immune cell expression in TGCTs is currently unknown and reports from literature regarding other types of cancer are scarce. Results from our cohort of 240 patients revealed that PD-L1 positive TILs are found more frequently in seminoma and embryonal carcinoma compared to other subtypes of TGCTs. These findings contribute to our earlier observations about prognostic value of PD-L1 expression in TGCTs tumor cells [23]. Seminoma as the most frequent histologic subtype with PD-L1 expressing TILs also proved to have the highest expression of PD-L1 on TILs. Moreover, for the first time we demonstrated the prognostic value of PD-L1 expressing TILs in TGCTs evidenced by significant difference in PFS and OS. The expression of PD-L1 on mononuclear cells found previously in urothelial carcinoma was associated with better prognosis, consistently with our findings in TGCTs [31]. The smallest number of TILs expressing PD-L1 were found in teratoma and choriocarcinoma. Accordingly, TILs in teratoma and choriocarcinoma expressed the lowest level of PD-L1, which could be one of mechanisms contributing to teratoma treatment resistance and aggressive biological behavior of choriocarcinoma. However, these subtypes were least frequent in the cohort, which could under-power our observations. Our data also proved the association of PD-L1 expressing TILs and prognostic groups according to IGCCCG. We observed lower expression in patients in poor risk category comparing to good and intermediate risk. Mediastinal lymphadenopathy, >3 metastatic sites, presence of NPVM and S3 stage were associated with low levels of PD-L1 expressed on TILs. Our findings suggest immune escape of tumor, mediated by decrease of PD-L1 expression on TILs in primary TGCTs. Tumors with low expression of PD-L1 on TILs seem to be easily facilitated towards disease dissemination and treatment response could be impaired as well. PD-L1 expression in tumor seems to correspond with PD-L1 expression on TILs in reverse manner, e.g. seminoma has the lowest expression of PD-L1 on tumor cells, but the highest expression of PD-L1 on TILs. Teratoma has the highest expression of PD-L1 on tumor cells, but the lowest expression on TILs [23]. In general, our previous and current study suggest association of poor prognostic features with higher PD-L1 expression in tumor and lower PD-L1 expression on TILs. Consistently, with our previous observation, there was lack of correlation between PD-1 expression on TILs and patients’ outcome [23]. The exact role of PD-L1 on TILs is not entirely clear owing to scarcity of the data in this field. Current mechanism of action can only be speculated and more research is necessary to adress this issue. Analysis of combined prognostic value of PD-L1 expression on tumor and TILs revealed four groups of patients with different prognosis patterns. PD-L1 seems to play different roles if found on tumor cells or TILs. Patients with high PD-L1 in tumor and low PD-L1 on TILs (as opposed to low PD-L1 in tumor and high PD-L1 on TILs) had the worst prognosis, therefore they could be candidates for novel treatment approaches. Better survival observed in patients with high PD-L1 on TILs despite high PD-L1 expression in tumor, suggested special biological value of PD-L1 expressing TILs positively altering a prognosis of TGCTs.

Testicular tissue is an immunologically privileged site with naturally suppressed immune responses mediated by PD-1: PD-L1 negative co-stimulation [32]. The role of PD-L1 in testicular immune privilege and homeostasis of immune responses has been therefore clearly identified [32]. However, cytotoxic T cells in tumor microenvironment are exposed to inhibition, thus providing immune escape for the tumor [8]. The efficacy of check-point inhibition in TGCTs is unknown. A result from 4 heavily pretreated patients treated with anti-PD-1 antibody have been recently published. Two patients with PD-1 negative primary mediastinal yolk sac tumors progressed immediatelly after anti-PD1 treatment. Third patient with PD-1 negative teratoma and yolk sac tumor had a mixed response with tumor marker decline after initial “pseudo progression”. Patient 4 with germinoma of the hypophysis strongly positive for PD-L1 achieved a durable near complete response with pembrolizumab and oral etoposide. PD-L1 expression on germ cell tumors may therefore play a predictive role for treatment with anti-PD1 agent. However, patient also received a concomitant treatment with oral etoposide, which could contribute to treatment efficacy of pembrolizumab [33]. Treatment with pembrolizumab in refractory TGCTs is currently under evaluation in a phase II study (NCT02499952). The association between PD-L1 expressing immune cells and treatment response to a check-point inhibitor have been recently explored in advanced urothelial cancer by Rosenberg et al. [34]. Increased levels of PD-L1 on immune cells were related to increased response to atezolizumab (anti-PD-L1 monoclonal antibody). Moreover, the immune activation gene subsets (CXCL9, CD8A) and other immune check-point genes (PD-L1, CTLA-4, TIGIT) were associated with immune cell PD-L1 expression, thus suggesting immune cell PD-L1 expression represents an adaptive immune regulation. Known data thus imply a PD-L1 inhibition could be a meaningful treatment target in TGCTs.

DAKO antibodies were used for the realization of this study. Previsously published studies have used various types of antibodies for immunohistochemical detection of PD1 and PD-L1 contributing to heterogenous scoring and results [34–36]. Therefore we suggest that staining results should always be interpreted in the context of the antibody used for PD1 and PD-L1 detection.

The study has some limitations, including the retrospective nature of the analysis, the absence of extragonadal germ cell tumors and the relative under-representation of choriocarcinoma and yolk sac tumor. Subpopulations of lymphocytes were not analyzed in this study. Another limiting factor is missing validation group of patients, thus the study should be considered as hypothesis generating.

In conclusion, this translational study is the first one to show significant differences in PD-L1 expressing TILs in different histological subtypes of TGCTs, as well as correlation between higher expression of PD-L1 on TILs in primary tumor and superior outcome in patients with primary TGCTs. PD-L1 expression on TILs is lower among patients with poor risk disease according to IGCCCG, suggesting immune escape and disease dissemination is mediated by decreased PD-L1 expression on TILs. Moreover, combined analysis of PD-L1 expression on tumor and TILs uncovered four distinct prognostic groups of patients. PD-1 expressing TILs did not have prognostic value in TGCTs nor did we find correlations between clinico-pathologic characteristics in our patient cohort. Based on these data we suggest PD-L1 expression on TILs of primary TGCTs is a novel prognostic marker and could be a potential therapeutic target. Further research is needed to determine its’ predictive value for tailoring anti-PD-L1 therapy in TGCTs.

## MATERIALS AND METHODS

### Experimental design

This retrospective translational study (Protocol IZLO1, Chair: M. Mego) included 240 patients with TGCTs treated from January 1999 to December 2013 in the National Cancer Institute of Slovakia, with available paraffin embedded tumor tissue specimen and sufficient follow-up clinical data. One hundred and thirty six patients (56.7%) in this study were also included in our previous report [23]. Patients with concurrent malignancy other than non-melanoma skin cancer in the previous 5 years were excluded. Data from all patients regarding age, tumor histology, clinical stage, type and number of metastatic sites, and delivery of systemic therapy were recorded and compared with PD1 and PD-L1 expression in tumor and TILs. The Institutional Review Board approved this retrospective study and a waiver of consent form for patients was granted.

### Tumor pathology

Pathology review was conducted at the Department of Pathology, Faculty of Medicine, Comenius University, by two pathologists (ZC and PB) associated with the study.

### Diagnosis and tissue samples

**Figure 3:**
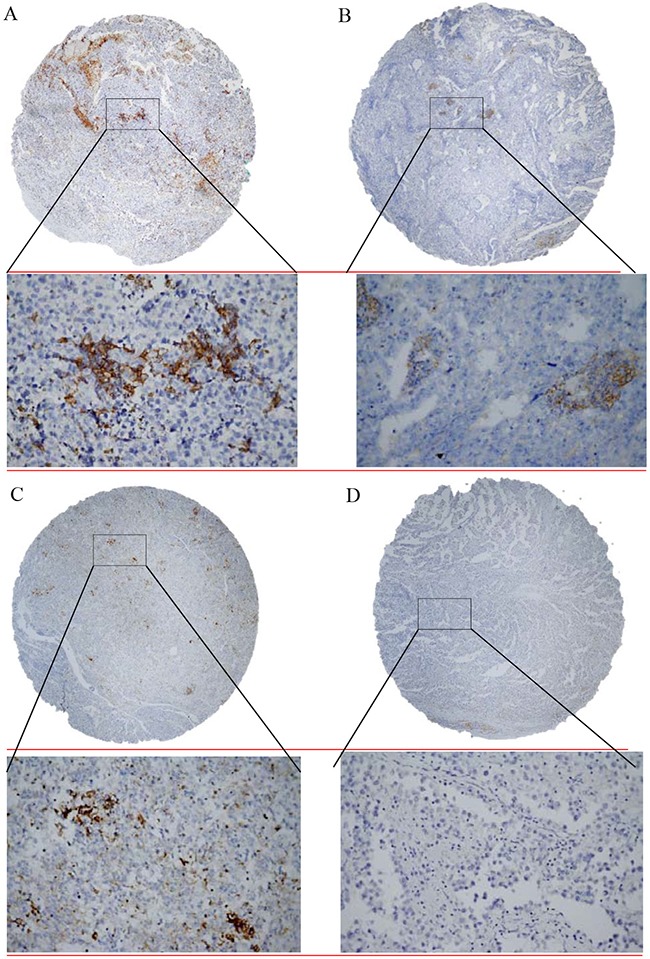
Immunohistochemical detection of programmed death-cell ligand 1 (PD-L1) expression in tumor infiltrating lymphocytes in testicular germ cell tumors **A.** Seminoma showed weak focal membranous PD-L1 positivity to negativity (brown colour) with strong cytoplasmic PD-L1 positivity of tumor infiltrating lymphocytes; **B.** Embryonal carcinoma with PD-L1 negativity and intermediate cytoplasmic PD-L1 positivity of tumor infiltrating lymphocytes; **C.** Yolk sac tumor with constant weak membranous PD-L1 positivity and strong cytoplasmic PD-L1 positivity of tumor infiltrating lymphocytes; **D.** Seminoma and tumor infiltrating lymphocytes negative for PD-L1. Original magnification ×40/x400.

The study included tumor specimens from 240 patients before administration of systemic therapy. All of these specimens were obtained from primary testicular tumors. The TGCTs were classified according to World Health Organization criteria [37]. PD1 and PD-L1 expression was evaluated in tumor, TILs and in lymphocytes between tubules adjacent to germ-cell neoplasia *in situ* (GCNIS) (Figure 3).

### Tissue microarray construction

According to tumor histology, one or two representative tumor areas from each histological subtype (e.g. 1-10 from each patient) of TGCTs were identified on H&E sections. Sections were matched to their corresponding paraffin blocks (the donor blocks), and 3-mm diameter cores of tissue were removed from these donor blocks with the multipurpose sampling tool Harris Uni-Core (Sigma-Aldrich, Steinheim, Germany) and inserted into the recipient master block. The recipient block was cut into 5-μm sections which were transferred to coated slides.

### Immunohistochemical staining

Slides were deparaffinized and rehydrated in phosphate buffered saline solution (10 mM, pH 7.2). The tissue epitopes were demasked using the automated water bath heating process in Dako PT Link (Dako, Glostrup, Denmark); the slides were incubated in TRIS-EDTA retrieval solution (10mM TRIS, 1mM EDTA pH 9.0) at 98°C for 20 minutes. The slides were subsequently incubated for 1 hour at room temperature with the primary mouse monoclonal antibody against PD-1 (Abcam, [NAT105]: AB52587) and rabbit monoclonal antibody against PD-L1 (Abcam [EPR1161(2)]: AB174838) diluted 1:100 in Dako REAL antibody diluent (Dako, Glostrup, Denmark) and immunostained using anti-mouse/anti-rabbit immuno-peroxidase polymer (EnVision FLEX/HRP, Dako, Glostrup, Denmark) for 30 minutes at room temperature, according to the manufacturer's instructions. Color reaction was developed with diaminobenzidine substrate-chromogen solution (DAB, Dako, Glostrup, Denmark) for 5 minutes. Finally, the slides were counterstained with haematoxylin, mounted and reacted for 5 minutes with diaminobenzidine substrate-chromogen solution (DAB, Dako, Glostrup, Denmark) for visualization. PD-1 and PD-L1 positivity of lymphocytes in the tonsil was used as a positive control, same tissue with omitting of the primary antibody served as negative control.

### Microscopy

Tissue samples were evaluated under a light microscope (Olympus BX40, Tokyo, Japan), the microphotographs were acquired using the camera (Nikon Instruments EOS1000D, Tokyo, Japan) and software DSLR Remote (Breezesys, Camberley, Surrey, UK).

### Immunohistochemical stain scoring

Tumor cores were independently assessed by two observers (ZC and PB) who were blinded to clinicopathological data. In cases of disagreement, the result was reached by consensus. TILs were identified in hematoxylin-eosin staining according to typical morphology (Figure 3). TILs with PD-1 and PD-L1 expressions were scored by weighted histoscore (HS) which accounts for both the extent of cell staining and staining intensity [38]. Briefly, the portion of positive cells was estimated on a scale from 0-100%. The average intensity of positively staining cells was given a score from 0 to 3 (0=no staining; 1=weak; 2= intermediate; and 3= strong staining). Weighted histoscore was then calculated by multiplying the percentage score by the intensity score, to yield a minimum value of 0 and a maximum value of 300. Based on the HS, PD-1 and PD-L1 expressions were graded as low (0–150) or high (160–300) as we described previously [39]. If multiple histologic subtypes were present in a sample, we chose the highest number among these subtypes for final PD-L1 expression of a mixed tumor.

### Statistical analysis

Patients’ characteristics were tabulated. A distri-bution of PD-1 and PD-L1 HS in TILs was significantly different from the normal distribution (Shapiro–Wilk test), therefore we used non-parametric tests for analyses. Analyses of differences in distributions of PD-1 and PD-L1 expression in TILs between the two groups of patients were performed using the Mann–Whitney U test, while Fisher's exact test or the χ2 test when appropriate were used, when PD-1 and PD-L1 expression was categorized as ‘low’ or ‘high’ according to the aforementioned criteria.

Median follow-up period was calculated as a median observation time among all patients and among those still alive at the time of their last follow-up. Progression-free survival (PFS) was calculated from the date of orchiectomy or the date of tumor biopsy to the date of progression or death or the date of last adequate follow-up. Overall survival (OS) was calculated from the date of orchiectomy or date of tumor biopsy to the date of death or last follow-up. PFS and OS were estimated using the Kaplan–Meier product limit method and compared by the log-rank test. A multivariable Cox proportional hazards model for PFS and OS was used to assess differences in outcome on the basis of PD1 and PD-L1 expression in tumor cells and TILs of primary tumor and prognosis according to IGCCCG (International Germ Cell Collaborative Group) [40]. All reported p values were two sided. For all statistical analyses, a p value <0.05 was considered as significant. Statistical analyses were performed using NCSS 2007 software (Hintze J, 2007, Kaysville, Utah, USA).

## SUPPLEMENTARY MATERIALS FIGURE


