# Supplementary material for: Prognostic role of programmed-death ligand 1 (PD-L1) expressing tumor infiltrating lymphocytes in testicular germ cell tumors
Source: Oncotarget. 2017 Feb 21;8(13):21794–805. doi: 10.18632/oncotarget.15585 (PMC5400624; doi:10.18632/oncotarget.15585)
Supplement: Supplementary file 1 [file oncotarget-08-21794-s001.pdf]

Prognostic role of programmed-death ligand 1 (PD-L1) expressing tumor infiltrating lymphocytes in testicular germ cell tumors

SUPPLEMENTARY FIGURE

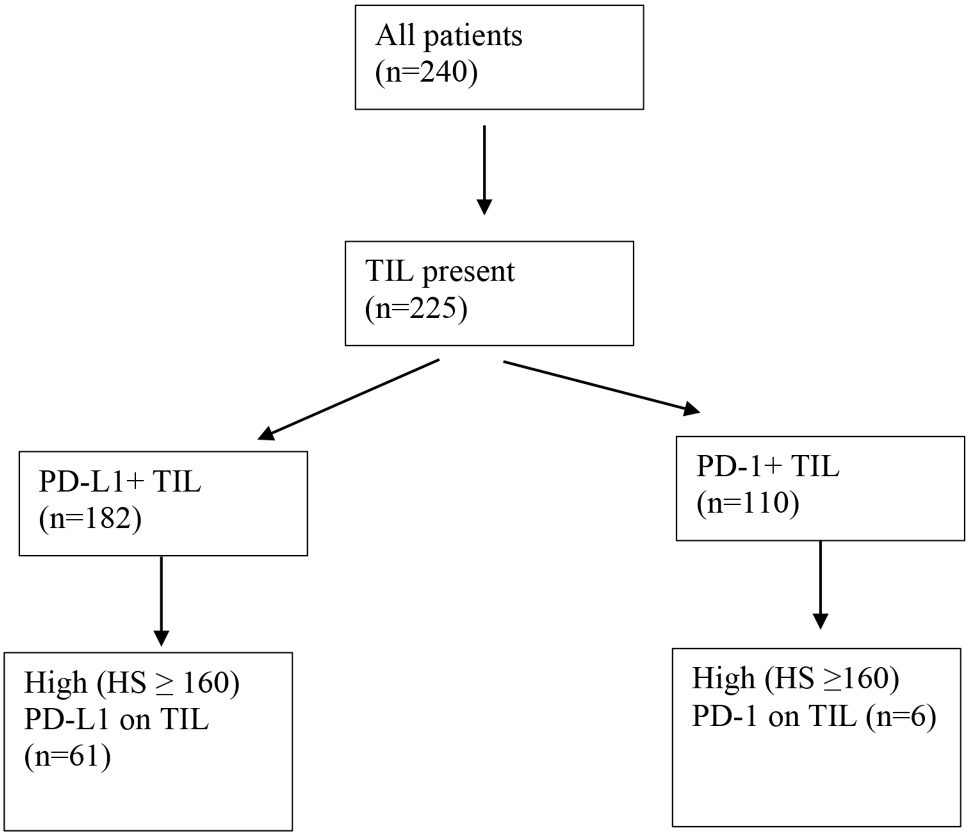

Supplementary Figure 1: Distribution of TILs among TGCT patients (n=240).
